# Inhibition of CRISPR-Cas9 ribonucleoprotein complex assembly by anti-CRISPR AcrIIC2

**DOI:** 10.1038/s41467-019-10577-3

**Published:** 2019-06-26

**Authors:** Annoj Thavalingam, Zhi Cheng, Bianca Garcia, Xue Huang, Megha Shah, Wei Sun, Min Wang, Lucas Harrington, Sungwon Hwang, Yurima Hidalgo-Reyes, Erik J. Sontheimer, Jennifer Doudna, Alan R. Davidson, Trevor F. Moraes, Yanli Wang, Karen L. Maxwell

**Affiliations:** 1 Department of Biochemistry, University of Toronto, 661 University Avenue, Suite 1600, Toronto, ON M5G 1M1 Canada; 2 Key Laboratory of RNA Biology, CAS Center for Excellence in Biomacromolecules, Institute of Biophysics, Chinese Academy of Sciences, Beijing, 100101 China; 3 University of Chinese Academy of Sciences, Beijing, 100049 China; 4 Department of Molecular Genetics, University of Toronto, 661 University Avenue, Suite 1600, Toronto, ON M5G 1M1 Canada; 5 Hefei National Research Center for Physical Sciences at the Microscale, School of Life Sciences, University of Science and Technology of China, Hefei, 230027 Anhui China; 6 Department of Molecular and Cell Biology, University of California, Berkeley, Berkeley, CA 94720 USA; 7 RNA Therapeutics Institute, University of Massachusetts Medical School, Worcester, MA 01605 USA; 8 Program in Molecular Medicine, University of Massachusetts Medical School, Worcester, MA 01605 USA; 9 Molecular Biophysics and Integrated Bioimaging Division, Lawrence Berkeley National Laboratory, Berkeley, CA 94720 USA; 10 Innovative Genomics Institute, University of California, Berkeley, CA 94704 USA; 11 Howard Hughes Medical Institute, University of California, Berkeley, CA 94720 USA; 12 Department of Chemistry, University of California, Berkeley, CA 94720 USA; 13 Gladstone Institutes, San Francisco, CA 94158 USA; 14 National Laboratory of Biomacromolecules, Institute of Biophysics, Chinese Academy of Sciences, Beijing, 100101 China

**Keywords:** X-ray crystallography, Bacteriophages, CRISPR-Cas systems

## Abstract

CRISPR-Cas adaptive immune systems function to protect bacteria from invasion by foreign genetic elements. The CRISPR-Cas9 system has been widely adopted as a powerful genome-editing tool, and phage-encoded inhibitors, known as anti-CRISPRs, offer a means of regulating its activity. Here, we report the crystal structures of anti-CRISPR protein AcrIIC2_*Nme*_ alone and in complex with Nme1Cas9. We demonstrate that AcrIIC2_*Nme*_ inhibits Cas9 through interactions with the positively charged bridge helix, thereby preventing sgRNA loading. In vivo phage plaque assays and in vitro DNA cleavage assays show that AcrIIC2_*Nme*_ mediates its activity through a large electronegative surface. This work shows that anti-CRISPR activity can be mediated through the inhibition of Cas9 complex assembly.

## Abstract (web summary)

Anti-CRISPR proteins offer the means of regulating CRISPR-Cas9 activity. Here the authors present the structure and biochemical characterisation of AcrIIC2_*Nme*_ alone and in complex with Cas9.

## Introduction

CRISPR-Cas systems provide adaptive immunity that protects bacteria and archaea against invasion by phages, plasmids, and other foreign genetic elements^1–3^. When a bacterial cell is invaded by a phage, the CRISPR-Cas system acquires a short segment of the phage genome and integrates it into the CRISPR locus where it can serve as a template for the production of mature CRISPR RNA (crRNA) molecules. These crRNAs form a complex with either a single protein effector or a multi-subunit effector complex that targets and degrades invading nucleic acids in a sequence-specific manner. CRISPR-Cas systems are divided into two classes, which can be further subdivided into six types and 33 subtypes, including their variants^4^. Class 1 systems (types I, III, and IV) form multi-subunit effector complexes, while Class 2 systems (types II, V, and VI) use a single protein to target invading genetic elements^5^. The type II protein, Cas9, has been widely adapted as a molecular tool for genome editing purposes.

In response to the evolutionary pressures posed by active CRISPR-Cas systems, phages have evolved protein inhibitors of these systems. The first described anti-CRISPR proteins were active against the type I-E and I-F systems in *Pseudomonas aeruginosa*^6–8^. Subsequently, anti-CRISPR proteins were identified against type II-C^9,10^, type II-A^11–13^, type I-D^14^, type I-C^15^, and type V-A^15,16^ CRISPR-Cas systems. The protein sequences of these anti-CRISPRs display high sequence diversity and the mechanisms by which they function also vary widely. Within the type I systems, anti-CRISPRs AcrIF1, AcrIF2, and AcrIF10 have been shown to interact directly with the Cascade complex and block DNA binding^17–21^. AcrIF3 interacts with the Cas3 nuclease and prevents its recruitment to the DNA-bound Cascade complex^22^, while AcrIF10 acts as a DNA mimic, binding to the basic residues that are critical for DNA binding. The mechanisms of activity of the type II anti-CRISPRs have proven to be similarly varied. Type II-C anti-CRISPR AcrIIC1 was shown to bind directly to the Cas9 HNH domain and prevent cleavage of the target DNA strand, while AcrIIC3 was shown to induce Cas9 dimerization and thereby inhibit DNA binding activity^23^. AcrIIA4 and AcrIIA2, which inhibit type II-A Cas9 proteins, were shown to occupy the PAM-interacting site, interacting with the RuvC, CTD, and TOPO domains of SpyCas9, and inhibiting the nuclease activity of SpyCas9 through multiple mechanisms^24^. Thus, previously characterized anti-CRISPRs function either through inhibition of nuclease activity or by blocking target DNA binding.

As Cas9 is a large multi-functional protein that mediates its activities through multiple domains, it provides a variety of surfaces that could potentially be targeted by anti-CRISPRs. Cas9 is composed of two lobes, the α-helical recognition (REC) lobe and the nuclease (NUC) lobe. The NUC lobe contains the HNH and RuvC endonuclease domains that are required for DNA cleavage activity, and the more variable PAM-interacting domain (PID). The two lobes are connected by the arginine-rich bridge helix. The PID is largely disordered in the apo-Cas9 structure, which prevents target DNA recognition in the absence of guide RNA. The transition of Cas9 to its active conformation requires binding of a guide RNA molecule^25,26^. This results in substantial structural rearrangements, with the most prominent conformational changes taking place in the REC lobe^27^. Binding to both target DNA and guide RNA are thought to be key regulators of Cas9 enzyme function^26^.

In this work, we investigate the mechanism of activity of anti-CRISPR protein AcrIIC2_*Nme*_. This 123-residue protein was previously shown to inhibit the activity of *Neisseria meningitidis* CRISPR-Cas9 in vivo and in vitro^9,23^. We show that AcrIIC2_*Nme*_ functions by inhibiting loading of the guide RNA molecule, thereby preventing formation of the active CRISPR-Cas9 surveillance complex. As previously characterized mechanisms of anti-CRISPR activity all target fully assembled CRISPR-Cas complexes, AcrIIC2_*Nme*_ provides a unique mechanism for anti-CRISPR activity.

## Results

### AcrIIC2_*Nme*_ binds to the bridge helix

**Fig. 1:**
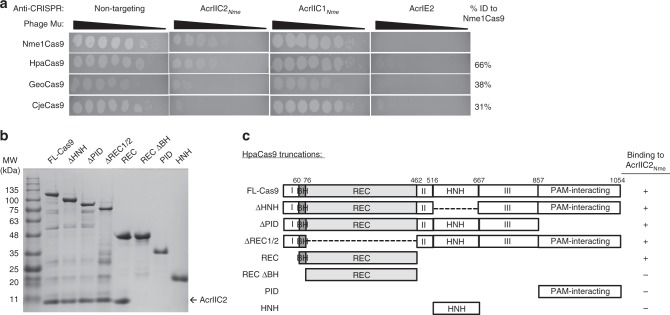
AcrIIC2_*Nme*_ inhibits Cas9 activity through an interaction with the bridge helix. **a** Plaquing of *E. coli* phage Mu targeted by type II-C Cas9 proteins (Nme1Cas9, HpaCas9, GeoCas9, CjeCas9) in the presence of AcrIIC2_*Nme*_, a type II-C anti-CRISPR with broad activity (AcrIIC1_*Nme*_) and a type I anti-CRISPR (AcrIE2). The sequence identity of the Cas9 proteins as compared to Nme1Cas9 is noted to the right of the figure. **b** Untagged anti-CRISPR was co-purified with 6x-His-tagged full-length HpaCas9, or domains thereof (**c**) using Ni-NTA affinity chromatography and the bound proteins were analyzed by SDS-PAGE and visualized using Coomassie staining. **c** Schematics of HpaCas9 truncations used to identify the domain with which AcrIIC2_*Nme*_ interacts. The bridge helix is denoted in dark gray, and the three sequence regions that comprise the RuvC domain are denoted as I, II, and III

We previously showed that anti-CRISPR protein AcrIIC2_*Nme*_ was able to robustly inhibit the cleavage activity of the *N. meningitidis* type II-C CRISPR-Cas9 protein^9,23^. As other anti-CRISPRs have been shown to have activity against multiple Cas9 orthologues^23^, we investigated the range of activity of AcrIIC2_*Nme*_ using an in vivo phage-targeting assay (Fig. 1a). In this assay, the Cas9 protein is expressed from a plasmid in *Escherichia coli* together with an sgRNA that targets *E. coli* phage Mu. This CRISPR targeting prevents phage Mu from forming plaques. In the presence of a functional anti-CRISPR protein, phage Mu is able to successfully infect the bacterial cell, leading to plaque formation. We determined that AcrIIC2_*Nme*_ was able to fully inhibit the activity of its cognate type II-C Cas9 protein from *N. meningitidis* (Nme1Cas9), as well as a homolog from *Haemophilus parainfluenzae* (HpaCas9) that shares 65% sequence identity^10^. By contrast, AcrIIC2_*Nme*_ showed very poor inhibitory activity against the type II-C Cas9 proteins from *Geobacillus stearothermophilus* (GeoCas9) and *Campylobacter jejuni* (CjeCas9), which share only 38% and 31% sequence identity with Nme1Cas9. These results are consistent with our previous work that showed AcrIIC2_*Nme*_ inhibits Nme1Cas9 and HpaCas9, but not more distantly related Cas9 proteins in vitro^10,23^.

To gain insight into how AcrIIC2_*Nme*_ inhibits Cas9 activity, we set out to identify the domain with which it interacts. Full-length Nme1Cas9 was susceptible to degradation in vivo and many of its isolated domains were insoluble. Thus, we used the closely related HpaCas9 for these studies because the holoenzyme and isolated domains were considerably more stable than Nme1Cas9. We co-expressed untagged AcrIIC2_*Nme*_ with 6-His-tagged HpaCas9 in *E. coli* and purified the resulting complex using Ni-affinity chromatography. AcrIIC2_*Nme*_ co-purified with Cas9, showing a specific interaction between the two proteins (Fig. 1b). We next tested for interactions with isolated Cas9 domains, including the HNH domain, the guide RNA recognition (REC) lobe, and the PID (Fig. 1c). We found that AcrIIC2_*Nme*_ co-eluted from the Ni-NTA column with the REC lobe (Fig. 1b). To further delineate the region of the REC lobe with which AcrIIC2_*Nme*_ interacts, we created a construct lacking the N-terminal arginine-rich bridge helix (REC-ΔBH). AcrIIC2_*Nme*_ was unable to stably interact with this domain. To determine if the bridge helix alone was sufficient for AcrIIC2_*Nme*_ binding to Cas9, we created a deletion mutant of Cas9 that maintained the bridge helix but lacked the REC1 and REC2 domains (ΔREC1/2). AcrIIC2_*Nme*_ still bound to this protein. Consistent with the bridge helix interaction, AcrIIC2_*Nme*_ did not bind to the isolated HNH or PID and was able to bind to Cas9 in their absence (Fig. 1b). These results indicate that the bridge helix is the primary binding site for AcrIIC2_*Nme*_.

### The interaction of AcrIIC2_*Nme*_ inhibits sgRNA binding

**Fig. 2:**
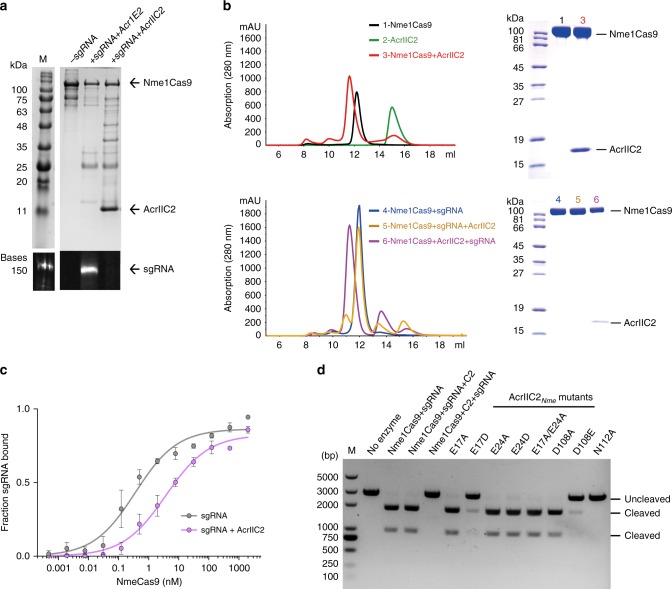
AcrIIC2_*Nme*_ inhibits sgRNA binding. **a** Purification of His-tagged Nme1Cas9 + sgRNA co-expressed with AcrIIC2_*Nme*_ or a type I anti-CRISPR protein (AcrIE2) using Ni-NTA chromatography. Analysis of the resulting elutions included SDS-PAGE followed by Coomassie staining (upper panel) and denaturing polyacrylamide/urea gel followed by SYBR Gold staining (lower panel). **b** Gel filtration chromatography shows that AcrIIC2_*Nme*_ interacts with sgRNA-free Nme1Cas9 (upper panel), but fails to bind to the Nme1Cas9-sgRNA complex (lower panel). The Nme1Cas9-sgRNA-AcrIIC2_*Nme*_ and Nme1Cas9-AcrIIC2_*Nme*_-sgRNA samples were reconstituted by incubation of purified Nme1Cas9, sgRNA, and AcrIIC2_*Nme*_ at a molar ratio of 1:1.3:4 on ice. Each component was added in the order listed, with an intermittent incubation of 30 min before adding the next component. All samples were fractionated on a Superdex 200 increase 10/300, and fractions between 11 and 13 mL were analyzed on SDS-PAGE. **c** Radiolabeled RNAs were incubated with increasing amounts of Nme1Cas9 in the absence (gray) or presence (purple) of AcrIIC2_*Nme*_, and the fraction of protein-bound RNA was determined by nitrocellulose filter binding. Source data are provided as a Source Data file. **d** DNA cleavage assays with Nme1Cas9 and AcrIIC2_*Nme*_. The components and order of addition are noted above each lane. The AcrIIC2_*Nme*_ mutants were mixed with Nme1Cas9 before adding the sgRNA

The Cas9 REC lobe mediates sgRNA binding^28^. To determine the effects of AcrIIC2_*Nme*_ on sgRNA binding, we co-expressed it in *E. coli* with His-tagged Nme1Cas9 and sgRNA and purified the resulting complex using affinity chromatography. AcrIIC2_*Nme*_ co-purified with Nme1Cas9, but no sgRNA was bound to the complex (Fig. 2a). By contrast, when Nme1Cas9-sgRNA was co-expressed with a type I-E anti-CRISPR protein, which does not inhibit Cas9, the sgRNA co-purified with Nme1Cas9 (Fig. 2a). Thus, the interaction of AcrIIC2_*Nme*_ with Nme1Cas9 appears to block sgRNA binding to Nme1Cas9. In addition, we observed increased proteolysis of Nme1Cas9 when it was co-expressed with AcrIIC2_*Nme*_ (Fig. 2a). Previous work has shown that the Cas9 apo protein binding to guide RNA results in conformational changes that render the protein more resistant to proteolysis^26,29,30^. These conformational changes are required to form the active complex for target DNA cleavage. The increased sensitivity of Cas9 to cellular proteases in the presence of AcrIIC2_*Nme*_ is consistent with its role in blocking sgRNA binding. To further probe whether the binding of AcrIIC2_*Nme*_ affects the assembly of the Nme1Cas9-sgRNA surveillance complex, we performed limited α-chymotrypsin proteolysis. Both apo-Nme1Cas9 and AcrIIC2_*Nme*_-bound Nme1Cas9 were sensitive to α-chymotrypsin, and they exhibited similar digestion patterns (Supplementary Fig. 1A). By contrast, Nme1Cas9 bound to sgRNA or sgRNA/target DNA showed increased resistance to proteolysis (Supplementary Fig. 1B). When apo-Nme1Cas9 was pre-incubated with AcrIIC2_*Nme*_ and then sgRNA or sgRNA/target DNA was added, the digestion patterns were similar to those observed for apo-Nme1Cas9, indicating that prior interaction with AcrIIC2_*Nme*_ blocked sgRNA binding (Supplementary Fig. 1C). Finally, size exclusion chromatography was used to verify that AcrIIC2_*Nme*_ forms a stable complex with Nme1Cas9 but is unable to interact with sgRNA-bound Nme1Cas9 complex (Fig. 2b). Taken together, these results imply that AcrIIC2_*Nme*_ inhibits Nme1Cas9 by disrupting the assembly of the Nme1Cas9-sgRNA complex.

To validate our observation that AcrIIC2_*Nme*_ inhibits the loading of sgRNA onto Nme1Cas9, we tested the ability of Nme1Cas9 to bind sgRNA in the absence and presence of AcrIIC2_*Nme*_ using a filter-binding assay. This revealed that addition of AcrIIC2_*Nme*_ at a four-fold molar ratio decreased the affinity of binding of sgRNA to Nme1Cas9 (Fig. 2c). Using isothermal calorimetry (ITC) we calculated binding affinities for AcrIIC2_*Nme*_ and sgRNA to apo-Nme1Cas9. We determined that AcrIIC2_*Nme*_ bound with a *K*_d_ of 200 nM, while the affinity of the sgRNA was ten times greater (*K*_d_ = 23 nM; Supplementary Fig. 2). When we added a two-fold molar excess of AcrIIC2_*Nme*_ to Nme1Cas9 before sgRNA addition, we found that the affinity of sgRNA binding decreased to 76 nM. When a 20-fold molar excess of AcrIIC2_*Nme*_ was added before sgRNA, the affinity of sgRNA binding decreased further, to 3.2 μM. Gel filtration chromatography was also used to analyze the equilibrium complexes formed under these reaction conditions. We found that some Nme1Cas9-sgRNA complex was present in the presence of a two-fold excess of AcrIIC2_*Nme*_, but not in the presence of 10-fold excess AcrIIC2_*Nme*_ (Supplementary Fig. 3A). These results indicate that AcrIIC2_*Nme*_ competes with sgRNA for the binding site on Nme1Cas9 and prevents formation of the active surveillance complex.

We next used in vitro competition assays to evaluate the competition between AcrIIC2_*Nme*_ and sgRNA for the binding site on Nme1Cas9. When sgRNA was mixed with Nme1Cas9, and then AcrIIC2_*Nme*_ was added to this pre-formed complex, there was no inhibition of DNA cleavage (Fig. 2d). When AcrIIC2_*Nme*_ was pre-bound to Nme1Cas9 at a ratio of 2:1 and then an equal amount of sgRNA was added, we found complete inhibition of DNA cleavage in vitro (Fig. 2d, Supplementary Fig. 3B). However, when sgRNA and AcrIIC2_*Nme*_ were added simultaneously, a two-fold excess of AcrIIC2_*Nme*_ did not appreciably inhibit Nme1Cas9 activity (Supplementary Fig. 3B). In fact greater than 10-fold excess of anti-CRISPR was required to block DNA cleavage activity under these conditions (Supplementary Fig. 3B). Collectively, these data show that the anti-CRISPR and sgRNA directly compete for the same binding site. AcrIIC2_*Nme*_ is able to interact with Nme1Cas9 and block its activity through disruption of sgRNA binding, however, it does not efficiently inhibit the activity of the pre-formed surveillance complex due to its weaker binding interaction with Nme1Cas9.

### AcrIIC2_*Nme*_ has an electronegative functional surface

**Fig. 3:**
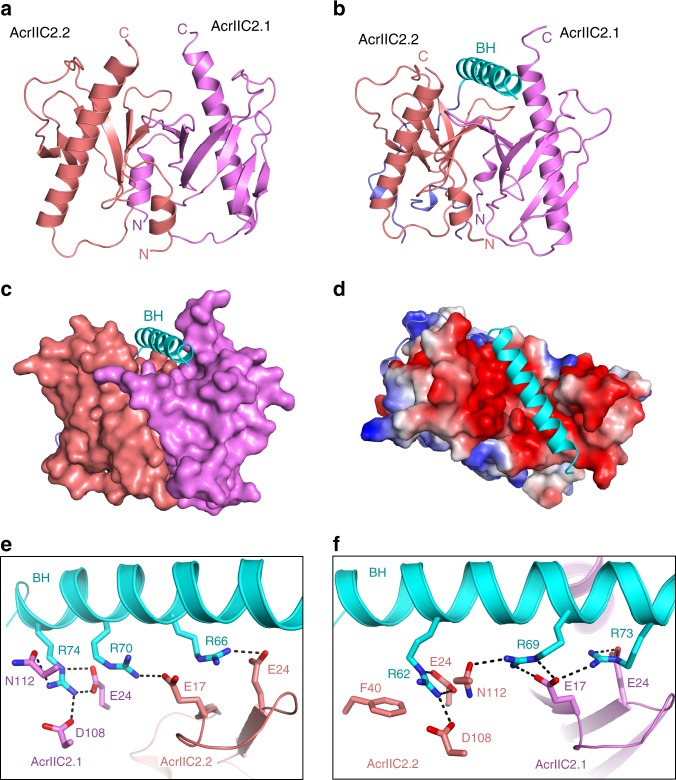
AcrIIC2_*Nme*_ functions as a homodimer. **a** The crystal structure of AcrIIC2_*Nme*_ solved by Se-SAD reveals a dimeric protein containing both α-helical and β-strand structural elements. Monomers are colored in violet and deep salmon, and the N- and C-termini are noted. **b** Cartoon representation of AcrIIC2-Nme1Cas9 complex. **c** Surface representation of AcrIIC2-Nme1Cas9 complex. **d** Electrostatic surface potential of AcrIIC2-Nme1Cas9 complex. **e**, **f** Interactions between the bridge helix and AcrIIC2_*Nme*_. The bridge helix is shown in cyan. AcrIIC2.1 and AcrIIC2.2 are shown in violet and deep salmon, respectively

To better understand the precise mechanism by which AcrIIC2_*Nme*_ inhibits Cas9 activity, we determined its crystal structure to a resolution of 2.5 Å using single-wavelength anomalous diffraction (SAD). All X-ray data collection and refinement statistics are summarized in Table 1. To date, the structures of 11 anti-CRISPR proteins have been determined [for review see ref. ^31^]. These anti-CRISPR families share no sequence identity and all display very different protein structures. Consistent with these previous observations, AcrIIC2_*Nme*_ shares no sequence or structural similarity with previously characterized anti-CRISPR proteins. A DALI search^32^ of the Protein Data Bank also did not reveal any significant similarity to any previously determined structure. The protein architecture of AcrIIC2_*Nme*_ consists of a six-stranded β-sheet composed of two anti-parallel β-strands followed by a Greek key motif, all wrapped around a 20-residue α-helix (Fig. 3a).

**Table 1:** Data collection and refinement statistics

	AcrIIC2	AcrIIC2	Se-AcrIIC2	AcrIIC2-Nme1Cas9	AcrIIC2-Nme1Cas9-proteolysis
**Data collection**					
PDB code	6N05	6JD7	–	6JDJ	6JDX
Space group	P4_3_2_1_2	C2	P6_3_22	P2_1_2_1_2_1_	P2_1_2_1_2_1_
Cell dimensions					
*a*, *b*, *c* (Å)	71.9, 71.9, 135.1	105.6, 73.5, 81.1	73.6, 73.6, 105.8	61.4, 77.4, 106.9	56.3, 77.4, 107.5
*α, β, γ* (°)	90.0, 90.0, 90.0	90.0, 129.8, 90.0	90.0, 120.0, 90.0	90.0, 90.0, 90.0	90.0, 90.0, 90.0
Wavelength (Å)	0.9789	0.97900	0.97918	0.97891	0.97894
Resolution (Å)	49.23–2.5 (2.59–2.50)	50.00–2.45 (2.49–2.45)	40.72–2.54 (2.58–2.54)	50.00–2.60 (2.64–2.60)	50–2.28 (2.32–2.28)
*R*_merge_	0.0979 (2.157)	0.074 (0.288)	0.138 (2.423)	0.154 (0.900)	0.087 (1.071)
Total reflections	181,759	90,912	214,497	198,730	235,170
Unique reflections	12,871	17,826	6026	16,424	21,775
*I*/σ*I*	23.8 (1.45)	19.7 (4.6)	21.4 (2.1)	18.5 (2.3)	26.6 (2.3)
Completeness (%)	99.9 (100)	99.7 (99.7)	99.9 (100)	99.9 (99.4)	99.1 (99.9)
Redundancy	14.1 (14.7)	5.1 (5.1)	35.6 (39.7)	12.1 (9.4)	10.8 (10.7)
**Refinement**					
Resolution (Å)	49.23–2.5	40.00–2.45		38.68–2.60	45.01–2.28
No. reflections	12,868	17,318		15,017	17,199
*R*_work_/*R*_free_	0.212/0.247	0.194/0.197		0.194/0.227	0.208/0.222
No. atoms					
Protein	1743	2781		2263	2226
Ligand/ion	0	5		0	4
Water	11	256		74	61
B-factors					
Protein	89.21	42.06		42.93	36.83
Ligand/ion		48.31			20.00
Water	65.45	40.11		40.18	32.61
R.m.s. deviations					
Bond lengths (Å)	0.008	0.020		0.005	0.019
Bond angles (°)	1.03	1.479		0.996	1.343

Values in parentheses are for highest-resolution shell

We also solved the crystal structure of AcrIIC2_*Nme*_ in complex with Nme1Cas9 to a resolution of 2.6 Å. We found that AcrIIC2_*Nme*_ bound to Nme1Cas9 in the same dimeric form as observed in the unbound state (Fig. 3b, c). Unexpectedly, only residues 16–77 of Nme1Cas9, corresponding to the bridge helix region and a partial fragment I of the RuvC domain, were observed in the complex (Supplementary Fig. 4A). Analysis of the AcrIIC2-Nme1Cas9 crystal by SDS-PAGE revealed that the rest of Nme1Cas9 was digested during crystallization. To confirm this finding, we treated the AcrIIC2-Nme1Cas9 complex with α-chymotrypsin protease, and then crystalized the digested complex. We solved this structure to a resolution of 2.3 Å and found that it was similar to the structure discussed above (Supplementary Fig. 4B and C). This further confirms that the bridge helix is the primary target for AcrIIC2_*Nme*_ activity.

We next analyzed interactions between AcrIIC2_*Nme*_ and Nme1Cas9. As shown in Fig. 3d, the AcrIIC2_*Nme*_ monomers form a dimer with a negatively charged surface on one side into which the arginine-rich bridge helix nestles. Four residues from each of the AcrIIC2_*Nme*_ monomers, E17, E24, D108, and N112, make interactions with the bridge helix (Fig. 3e, f). In AcrIIC2.1, the side-chain of E17 hydrogen bonds with Nme1Cas9 residues R69 and R73, E24 interacts with residues R73 and R74, while D108 and N112 form hydrogen bonds with the side-chain of R74. Similarly, the side-chains of E24, D108, E17, and N112 of AcrIIC2.2 interact with Nme1Cas9 residues R62, R66, R69, and R70.

**Fig. 4:**
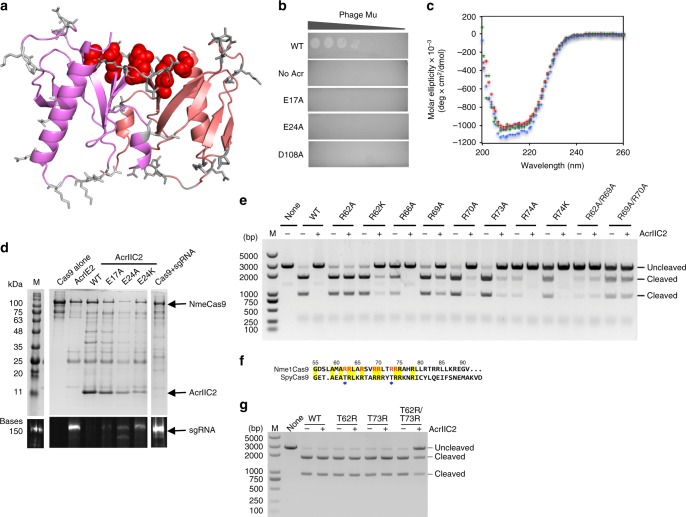
AcrIIC2_*Nme*_ activity is mediated through a large electronegative surface. **a** AcrIIC2_*Nme*_ surface-exposed residues targeted for site-directed mutagenesis are shown on the surface of the protein. The side chain positions at which amino acid substitutions did not affect activity are shown in gray, while the three residues that showed the large decrease in activity when substituted are shown in red. **b** Representative results of the in vivo phage plaque assay. Serial dilutions of phage Mu plated on *E. coli* reveal that E17A, E24A, and D108A mutants have lost the ability to inhibit CRISPR-Cas9. **c** Representative circular dichroism spectroscopy scans of wild type anti-CRISPR (red) and three inactive mutants (E17A, blue; E24A, green; D108A, purple) show that the inactive mutants maintain their secondary structure. **d** Co-purification of AcrIIC2_*Nme*_ mutants with 6x-His-tagged Nme1Cas9 reveals decreased binding of the inactive anti-CRISPRs to Nme1Cas9, and corresponding increase in the amount of Cas9-bound sgRNA. **e** In vitro DNA cleavage assays with wild type Nme1Cas9 and the site-directed mutants in the bridge helix domain in the presence and absence of AcrIIC2_*Nme*_. **f** Sequence alignment of the bridge helix of Nme1Cas9 and SpyCas9. The residue numbers of Nme1Cas9 are shown above the sequence. The amino acids that are identical in both Nme1Cas9 and SpyCas9 are shown with yellow background. The Nme1Cas9 amino acids that interact with AcrIIC2_*Nme*_ are shown in red. Residues T62 and T73 of SpyCas9 are highlighted by blue stars. **g** DNA cleavage assay with the wild type or mutant SpyCas9 either in the presence or absence of AcrIIC2_*Nme*_ to test whether AcrIIC2_*Nme*_ protein inhibits the activity of SpyCas9 mutants

To determine if the interactions between AcrIIC2_*Nme*_ and the bridge helix of Nme1Cas9 are required and sufficient for anti-CRISPR inhibitory activity, we mutated 19 residues distributed widely across the surface of AcrIIC2_*Nme*_ (Fig. 4a and Table 2). We also created a mutant lacking the final 12 amino acids (Δ112–123), which were not resolved in one of the apo crystal structures. The anti-CRISPR activity of each mutant was tested using the in vivo phage-targeting assay, in which expression of wild type AcrIIC2_*Nme*_ inactivates Nme1Cas9 and allows phage Mu to plaque. Mutation of three of the four amino acids that make direct contacts with the bridge helix (E17A, E24A, D108A) displayed a complete lack of anti-CRISPR activity in vivo (Fig. 4b and Table 2) while the other mutants showed changes in activity of less than 10-fold. Deletion of residue N112 (Δ112–123 mutant) did not affect activity. In vitro DNA cleavage assays further verified these results. Substitution of E17, E24, and D108 with Ala severely inhibited the activity of AcrIIC2_*Nme*_, as did the variant E24D (Fig. 2d). By contrast, mutants that maintained the negative charge at two of these positions (E17D and D108E) had similar levels of inhibition to that of wild type AcrIIC2_*Nme*_. Circular dichroism spectroscopy of the three mutants with abrogated in vivo activity revealed spectra similar to the wild type protein (Fig. 4c), showing that the lack of activity was not due to a folding defect. In addition, cooperative thermal denaturation curves and melting temperatures similar to the wild type protein (Table 3) indicated that these mutants maintained stable, folded structures. We conclude that the negatively charged surface of AcrIIC2_*Nme*_ and the positively charged Nme1Cas9 bridge helix comprise a critical interaction interface that is required for anti-CRISPR activity.

**Table 2:** In vivo phage plaquing assays

Residue substitution	Fold reduction in titre
K9A	<10
E17A	10^7^
R19A	<10
E21A	<10
N22A	<10
E24A	10^7^
E24K	<10
D42A	<10
T45A	<10
E47A	<10
K51A	<10
P52A	<10
E62A	<10
R64A	<10
N84A	<10
N85A	<10
K86A	<10
K88A	<10
E91A	<10
D108A	10^7^
D108E	<10
Δ112–123	<10

**Table 3:** Thermal stability values (*T*_m_) derived by circular dichroism spectroscopy

AcrIIC2 construct	*T*_m_ (°C)
WT	55.7 ± 0.6
E17A	59.7 ± 0.8
E24A	59.0 ± 0.9
D108A	61.8 ± 0.5

Source data are provided as a Source Data file

To further validate this functional surface, we assayed the ability of non-functional AcrIIC2_*Nme*_ mutants to inhibit sgRNA binding. We co-expressed His-tagged Nme1Cas9 with its sgRNA from a plasmid in *E. coli* and introduced a second plasmid that expressed either wild type AcrIIC2_*Nme*_, one of the inactive mutants, or a type I-E anti-CRISPR protein. Using nickel affinity chromatography we purified Nme1Cas9 and the associated sgRNA and anti-CRISPR proteins. In the absence of anti-CRISPR or in the presence of a control anti-CRISPR that targets the type I-E system, sgRNA co-purified with Nme1Cas9 (Fig. 4d). In the presence of wild type AcrIIC2_*Nme*_, no sgRNA co-purified, confirming the ability of this anti-CRISPR to block sgRNA binding in vivo. The inactive mutants showed varying levels of binding to Nme1Cas9, resulting in varying levels of inhibition of sgRNA binding (Fig. 4d). E17A was severely compromised, while E24A showed some inhibition of sgRNA binding, but not as strong as the wild type anti-CRISPR protein. Interestingly, both E24A and D108A maintained the ability to bind to Nme1Cas9, but were outcompeted by the sgRNA when it was added to the pre-formed AcrIIC2_*Nme*_-Nme1Cas9 complex (Supplementary Fig. 5A,B). Moreover, a double mutant, E17A/E24A, was completely outcompeted by the sgRNA (Supplementary Fig. 5C). These results further confirm that the acidic residues of AcrIIC2_*Nme*_ are crucial for the inhibition of Nme1Cas9.

We next examined residues in the Nme1Cas9 bridge helix that interact with AcrIIC2_*Nme*_ in the crystal structure. We first targeted position R62 and showed that substitution with Ala decreased inhibition of Nme1Cas9 by wild type AcrIIC2_*Nme*_. By contrast, substitution with Lys, which maintains the charge interaction with residue E24 in AcrIIC2_*Nme*_, allowed the anti-CRISPR to retain its inhibitory activity (Fig. 4e). These data suggest that the interaction between R62 of Nme1Cas9 and E24 of AcrIIC2.2 is crucial for inhibition. Similarly, mutation of position R69 in the bridge helix of Nme1Cas9, which slightly decreased DNA cleavage activity, dramatically reduced the inhibitory activity of AcrIIC2_*Nme*_. Substitution of four other positively charged residues in the bridge helix (R66A, R70A, R73A, and R74A) had little effect on the inhibitory activity of AcrIIC2_*Nme*_ (Fig. 4e). Together, these results indicate that the interactions between AcrIIC2_*Nme*_ and the bridge helix of Nme1Cas9 are essential for inhibition.

To determine if the sequence of the bridge helix is sufficient for inhibition by AcrIIC2_*Nme*_, we compared the amino acid sequence of Nme1Cas9, which is robustly inhibited by AcrIIC2_*Nme*_, with SpyCas9, which is not inhibited. We identified two positions in the SpyCas9 bridge helix (T62 and T73) that are arginine residues in Nme1Cas9 (Fig. 4f). We substituted these positions singly and in combination, and discovered that the in vitro DNA cleavage activity of the SpyCas9 double mutant was inhibited by AcrIIC2_*Nme*_ (Fig. 4g). This further confirms the importance of the interaction between AcrIIC2_*Nme*_ and the bridge helix of Cas9. It also emphasizes a potential role for anti-CRISPRs in driving Cas9 evolution.

### AcrIIC2_*Nme*_ blocks binding of sgRNA stem loops 1 and 2

**Fig. 5:**
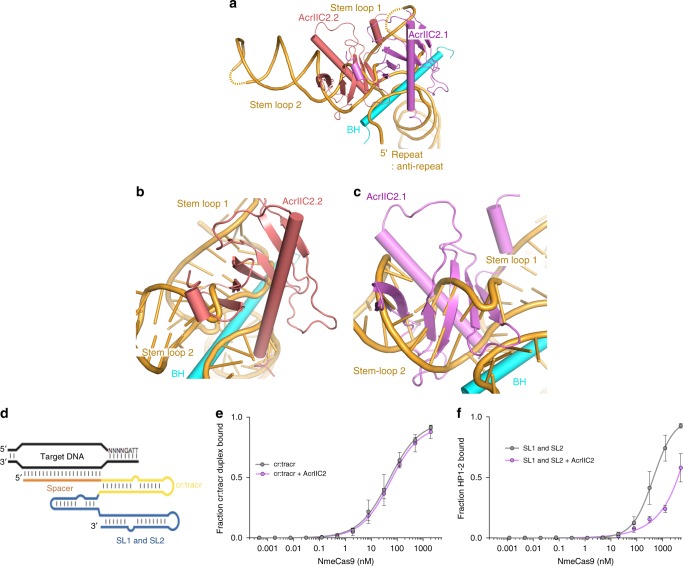
AcrIIC2_*Nme*_ blocks binding of the sgRNA by occluding the binding sites for stem loops 1 and 2. **a** Structural comparison between AcrIIC2_*Nme*_-Nme1Cas9 and Nme1Cas9-sgRNA binary complex based on the superimposition of bridge helix regions. Monomer AcrIIC2.2 occupies the major groove where stem loop 2 should bind (**b**) while AcrIIC2.1 occupies the positions of stem loop 1 and the seed region (**c**). **d** Schematic sgRNA, indicating the repeat:anti-repeat (cr:tracr) and 3′ stem loop 1 and 2 (SL1, SL2). Radiolabeled sgRNA truncations were incubated with Nme1Cas9-AcrIIC2_*Nme*_ complex and the fraction of protein-bound RNA was determined by nitrocellulose filter binding when only the cr:tracr (**e**) or 3′ stem loops 1 and 2 (**f**) were present. Source data are provided as a Source Data file

To further establish the impact of AcrIIC2_*Nme*_ binding on the assembly of Nme1Cas9 with sgRNA, we compared the structure of AcrIIC2_*Nme*_ bound to Nme1Cas9 with the Nme1Cas9-sgRNA binary complex by aligning the bridge helix regions. The structural superposition shows that two AcrIIC2_*Nme*_ monomers make major clashes with stem loops 1 and 2, and slightly overlap the seed region of the sgRNA (Fig. 5a). Monomer AcrIIC2.2 occupies the major groove of stem loop 2, displacing the duplex formed by nucleotides 125–131:98–90 and the adjacent single-stranded region at the 5′-end of the sgRNA (Fig. 5b). Monomer AcrIIC2.1 occupies the positions of nucleotides 84–87 and seed region from nucleotides 20–23 (Fig. 5c). To confirm the importance of these interactions, we assessed the ability of truncated sgRNA constructs to bind Nme1Cas9 in the presence of AcrIIC2_*Nme*_. We found that the 5′ cr:tracr duplex bound to Cas9 with equal affinities in the presence and absence of AcrIIC2_*Nme*_ (Fig. 5d, e). By contrast, the interaction of the 3′ stem loops was inhibited in the presence of AcrIIC2_*Nme*_ (Fig. 5d, f). These terminal stem loops have been shown to assist in stabilizing the sgRNA and supporting stable complex formation with the Cas9 protein^33^. These results confirm that AcrIIC2_*Nme*_ interferes with sgRNA binding through an interaction that blocks the binding site of the stem loops 1 and 2.

## Discussion

In this work, we investigated the mechanism of activity of anti-CRISPR protein AcrIIC2_*Nme*_. We found that it blocks Cas9 binding to sgRNA, thereby inhibiting biogenesis of the surveillance complex. This interference is mediated through an interaction with the arginine-rich bridge helix, which connects the REC lobe to the NUC lobe. High-resolution studies of Cas9-sgRNA complexes with and without target DNA bound show that the bridge helix contacts the sgRNA, in particular the region that pairs with nucleotides nearest the PAM sequence in the target DNA^28,34^. The crystal structure of AcrIIC2_*Nme*_ revealed a highly negatively charged surface that binds to the positively charged bridge helix. Mutation of this electronegative surface decreases the affinity of AcrIIC2_*Nme*_ for Cas9, resulting in loss of anti-CRISPR activity. The bridge helix, which forms part of the endonuclease functional core, is a universal feature of Cas9 proteins^26^ and thus provides a reliable target for inhibition by anti-CRISPR proteins. The targeting of a highly conserved domain by AcrIIC2_*Nme*_ is similar to that observed for AcrIIC1_*Nme*_, which was shown to target the HNH endonuclease domain for inhibition^23^.

Other previously characterized type I and type II anti-CRISPRs revealed two general mechanisms for blocking CRISPR-Cas activity. The most common mechanism observed to date is inhibition of DNA binding through a direct interaction with the CRISPR surveillance complex. The second mechanism is blocking DNA cleavage by inhibiting nuclease activity, either through interaction with a Cas9 endonuclease domain^23^ or the Cas3 endonuclease protein in type I systems^17,22^. The mechanism of activity for AcrIIC2_*Nme*_ that we determined in this study, disruption of CRISPR-Cas surveillance complex assembly through an interaction with the bridge helix, is in agreement with the study recently published by Zhu et al. in which they examined mechanisms of inhibition for anti-CRISPR proteins AcrIIC2_*Nme*_ and AcrIIC3_*Nme*_^35^. Like Zhu et al., we found that pre-binding sgRNA to Nme1Cas9 greatly reduced the ability of AcrIIC2_*Nme*_ to interact with the complex and inhibit DNA cleavage activity due to its much lower affinity for Cas9 as compared to the sgRNA. Using structural and biochemical analyses, we showed that AcrIIC2_*Nme*_ competes for the binding site of the sgRNA stem loops 1 and 2. In the natural CRISPR-Cas9 system, where the crRNA and tracrRNA are separate molecules instead of a single fused molecule as in the case of the sgRNA, AcrIIC2_*Nme*_ may compete more effectively with the tracrRNA for binding to Cas9. This might endow the anti-CRISPR with an increased ability to disrupt pre-formed surveillance complexes that are present in the cell when the phage infects.

In addition to blocking the surveillance complex formation, the activity of AcrIIC2_*Nme*_ leaves the Cas9 protein trapped in its apo form, which is much more sensitive to the activity of cellular proteases than are the RNA- and DNA-bound forms. Thus, the introduction of this anti-CRISPR into a cell may lead to a decrease in the steady state levels of full-length Cas9 in the cell, providing an additional mechanism of anti-CRISPR activity. This activity of AcrIIC2_*Nme*_ may explain the differences we observed in the in vivo and in vitro assays. When AcrIIC2_*Nme*_ was co-expressed with Nme1Cas9 in vivo and the resulting complex was purified, no sgRNA was found to be associated with the complex (Fig. 2a). This contrasted with in vitro experiments, in which a great excess of anti-CRISPR was required to inhibit sgRNA binding. AcrIIC2_*Nme*_ is robustly produced, soluble to high concentration, and resistant to bacterial proteases in *E. coli*. It accumulates to high levels in the cell that allow it to bind to Nme1Cas9 immediately as the Cas9 protein is being produced. The sgRNA, by contrast, is probably less stable in the cell due to the activity of cellular nucleases. For these reasons, the anti-CRISPR likely has a larger competitive advantage in vivo as compared with the purified in vitro system. The reduction of Nme1Cas9 steady-state levels in the presence of AcrIIC2_*Nme*_ was previously observed in mammalian cells^10^, and probably also contributes to the ability of AcrIIC2_*Nme*_ to efficiently inhibit genome editing in these cells. Other anti-CRISPRs that inhibit DNA binding or interfere with nuclease activity do not appear to share this type of two-pronged inhibitory mechanism. Additionally, the activity of AcrIIC2_*Nme*_ may also serve to inhibit spacer acquisition, which has been shown to be Cas9-dependent^36,37^.

Inhibiting the formation of the active CRISPR-Cas9 surveillance complex intuitively seems that it would not provide an advantage to infecting phages, as they could not overcome the pre-assembled surveillance complexes. However, recent work has shown that multiple phages need to infect a cell in order to provide a critical mass of anti-CRISPR protein to overwhelm the CRISPR system^38,39^. Even anti-CRISPRs that inhibit fully formed surveillance complexes fail to inactivate the CRISPR-Cas system if the phage population numbers fall below a critical threshold. When phages infect but fail to replicate in cells with active CRISPR systems, they still produce small amounts of anti-CRISPR proteins that leave the bacterial cell in an immunocompromised state. Thus, AcrIIC2_*Nme*_ molecules produced by a phage that was ultimately destroyed by Cas9 could persist within the cell and inhibit CRISPR-Cas complexes assembled after the infection. This would, in turn, increase the likelihood of successful replication by the next infecting phage. The expression of AcrIIC2_*Nme*_ would also be an effective means for a prophage to keep the CRISPR system turned off once it integrated into the host genome. Thus, a phage encoding an anti-CRISPR that prevents assembly of the CRISPR-Cas9 complex may not be at an evolutionarily disadvantage to one that inhibits the pre-formed complex. The distinct mechanism of activity of AcrIIC2_*Nme*_, inhibiting assembly of a CRISPR-Cas complex, further emphasizes the amazing diversity of inhibitors that phages have evolved to counteract the existential challenge posed by CRISPR-Cas systems.

## Methods

### Plasmid construction

Plasmids encoding the Cas9 proteins used in the in vivo phage-targeting assay were generated by Gibson assembly using primers listed in Supplementary Table 1. The pGeoCas9-sgRNA plasmid^23^ was used as the starting vector. GeoCas9 and its sgRNA were replaced with the variant Cas9 proteins and their corresponding sgRNAs (sequences listed in Supplementary Table 1). The sgRNAs were synthetized as part of gblock fragments (IDT) along with overhangs to clone using Gibson assembly. All fragments used in the assembly reactions were first amplified using Phusion High-Fidelity DNA Polymerase (Thermo Fisher Scientific). The reactions were prepared according to manufacturer’s recommendations and 2 μL of the assembly reaction solution were transformed in High Efficiency chemically competent *E. coli* cells (New England Biolabs). Clones were screened by restriction digestion and were sequence-verified. The Cas9-encoding plasmids were linearized with BsaI and ligated to DNA encoding crRNA targeting phage Mu, which were generated by annealing of two complementary oligonucleotides carrying overhanging BsaI ends.

For expression and protein purification in *E. coli*, NcoI-HindIII DNA inserts encoding wild type and mutant AcrIIC2_*Nme*_ proteins from pCDF-1b plasmids were sub-cloned into a pHAT4^40^ expression plasmid such that the protein was expressed with an N-terminal 6-His tag. The boundaries for the HpaCas9 HNH, PID, and REC domains were determined by alignment with GeoCas9, whose domain boundaries were previously described^23^. These domains were cloned into expression plasmid pCDF-1b with a 6-His tag fused to their C-termini. Point mutations in *acrIIC2*_*Nme*_ were generated by site-directed mutagenesis. The desired nucleotide mutations were introduced in the middle of 40 bp complementary primers (Supplementary Table 1). Sixteen PCR cycles were performed using Phusion^®^ High-fidelity DNA polymerase (ThermoScientific), and the PCR products were treated with DpnI endonuclease. The sample was ethanol precipitated and transformed into DH5α cells. Plasmids were isolated and mutations confirmed by sequencing.

### Phage plaque assays

Plasmids expressing the different Cas9 proteins containing a spacer targeting phage Mu were co-transformed in BB101 cells with a plasmid expressing wild type or mutant AcrIIC2_*Nme*_. Cells containing both plasmids were subcultured in Lysogeny broth (LB) supplemented with chloramphenicol and streptomycin and grown for 2 h, at which point anti-CRISPR expression was induced with 0.01 mM IPTG for 3 h. 200 μL of cells were mixed with soft agar and top-plated on LB supplemented with both antibiotics and 200 ng mL^−1^ aTc, 0.2% arabinose, and 10 mM MgSO_4_. Serial dilutions of phage Mu were spotted on top and the plates were incubated overnight at 37 °C. Experiments were performed in triplicate, with representative replicates shown in the figure panels.

### Cas9-AcrIIC2_*Nme*_ pull-down experiments

*E. coli* BL21 cells were co-transformed with 6-His-tagged HpaCas9 constructs in a pMCSG7 backbone^10^, and a pCDF-1b vector encoding untagged AcrIIC2_*Nme*_. Cells were grown in Terrific Broth (TB) at 37 °C to an optical density of 0.8. Protein expression was induced by the addition of 1 mM IPTG for 18 h at 16 °C. Cells were lysed by sonication in binding buffer (50 mM Tris pH 7.5, 200 mM NaCl, 5% Glycerol, 20 mM Imidazole). Clarified lysates were bound to Ni-NTA agarose (Qiagen) for 30 min at 4 °C, washed with binding buffer supplemented with 30 mM imidazole and bound protein was eluted with buffer containing 300 mM imidazole. 6-His-tagged Nme1Cas9 + sgRNA^9^ ribonucleoprotein with wild type or mutant AcrIIC2_*Nme*_ was purified using the same protocol. Complexes were analyzed by SDS-PAGE on a 15% Tris–Tricine gel and visualized by Coomassie staining. The amount of bound sgRNA was examined using a 12.5% polyacrylamide/urea gel and visualized by SYBR Gold (Thermo Fisher Scientific) staining. Experiments were performed in triplicate, with representative replicates shown in the figure panels.

### Filter binding assays

Filter binding was performed as described previously^23^. Filter binding was conducted in 1× Binding Buffer (20 mM Tris, pH 7.5, 100 mM KCl, 5 mM MgCl_2_, 1 mM DTT, 5% (vol/vol) glycerol, 0.01% Igepal CA-630, 10 μg mL^−1^ yeast tRNA, and 10 μg mL^−1^ BSA). Nme1Cas9 was mixed with a 4× molar ratio of AcrIIC2_*Nme*_ at the maximum concentration in Binding Buffer and was diluted in 1× Binding Buffer. Less than 0.01 nM radiolabeled sgRNA or the indicated guide truncation was added and allowed to incubate for 30 min at 37 °C. During incubation Tufryn (Pall Corporation), Protran (Whatman), and Hybond-N+ (GE Healthcare) membranes were soaked in Binding Buffer omitting the detergent, yeast tRNA and BSA and arranged on a dot blot apparatus above two layers of Whatman paper. The complexing reactions were loaded onto the dot blot apparatus and vacuum was applied. The membranes were dried and visualized by phosphorimaging and quantified using ImageQuant. All experiments were carried out in triplicate, with averaged values shown in the figure panels. Data were fit to a binding isotherm using Prism (GraphPad Software), and *K*_d_ values of 0.24 ± 0.14 nM for sgRNA alone, and 2.2 ± 1.3 nM in the presence of AcrIIC2_*Nme*_ were determined.

The sgRNA sequences for the truncation binding experiments are as follows:

Full length sgRNA—GGTCTGTAAGCGGATGCCATATGGTTGTAGCTCCCTTTCTCGAAAGAGAACCGTTGCTACAATAAGGCCGTCTGAAAAGATGTGCCGCAACGCTCTGCCCCTTAAAGCTTCTGCTTTAACGGGCTT

Repeat:anti-repeat—GGTTGTAGCTCCCTTTCTCGAAAGAGAACCGTTGCTACAATAA

SL1 and SL2—GGCCGTCTGAAAAGATGTGCCGCAACGCTCTGCCCCTTAAAGCTTCTGCTTTAACGGGCTT

### Isothermal titration calorimetry

Nme1Cas9, AcrIIC2_*Nme*_, sgRNA and (Nme1Cas9-AcrIIC2_*Nme*_) complexes of 1:2, 1:10, and 1:20 molar ratios were extensively dialyzed against a buffer containing 300 mM NaCl, 20 mM Tris, pH 7.5. Titrations were performed using a MicroCal PEAQ-ITC system (Malvern Instruments) at 20 °C. To determine the affinity of binding for Nme1Cas9 and AcrIIC2_*Nme*_, 100 μM AcrIIC2_*Nme*_ was titrated into 10 μM Nme1Cas9. To determine the affinities of sgRNA in the presence and absence of AcrIIC2_*Nme*_, 90 μM Nme1Cas9 with or without AcrIIC2_*Nme*_ was titrated with 10 μM sgRNA. The resulting ITC curves were processed using Origin 7.0 software (OriginLab) using the “One Set of Sites” fitting model. Experiments were carried out in triplicate, with representative replicates shown in the figure panels.

### Competitive binding assays

AcrIIC2_*Nme*_, sgRNA, and Nme1Cas9 (molar ratios of 2:1:1, 3:1:1, or 10:1:1) were mixed together on ice and then centrifuged at 13,800×*g* at 4 °C for 10 min. The samples were loaded into a Superdex 200 Increase 10/300 (GE Healthcare) column for separation using gel filtration chromatography. The buffer used for gel filtration contained 300 mM NaCl, 20 mM Tris, pH 7.5.

### In vitro DNA cleavage assays

The pUC19 target DNA plasmid (35 bp target DNA cloned into the pUC19 vector) was linearized by ScaI digestion before the cleavage reactions. In the presence or absence of AcrIIC2, the Cas9-sgRNA complex was incubated with 300 ng pUC19 target DNA in 10 μL reaction buffer containing 20 mM Tris–HCl pH 7.5, 100 mM KCl, 10 mM MgCl_2_, 1 mM DTT and 5% glycerol. All reactions were stopped by adding 1 μL 0.5 M EDTA and 1 μL 0.1 mg mL^−1^ Proteinase K for 30 min at room temperature. The reaction products were run on 1% agarose gels, and gels were stained with ethidium bromide for product detection. All experiments were carried out at least in triplicate, with representative replicates shown in the figure panels.

### Competitive cleavage assays

For the competition cleavage assays, two incubation methods were employed to prepare the samples for DNA cleavage. First, Nme1Cas9 was incubated with AcrIIC2_*Nme*_ at molar ratios of 1:0, 1:2, 1:4, 1:10, 1:20, and 1:40 on ice for 15 min, 1.1 fold of sgRNA was added and the samples were incubated for an additional 15 min. Second, 1.1 fold of sgRNA was simultaneously mixed with AcrIIC2_*Nme*_ and Nme1Cas9 and incubated for 15 min on ice. The resulting enzyme complexes were incubated with 300 ng pUC19 target DNA in 10 μL reaction buffer (20 mM Tris–HCl pH 7.5, 100 mM KCl, 10 mM MgCl_2_, 1 mM DTT and 5% glycerol). The reactions were allowed to proceed at 37 °C for 10 min, and were stopped by the addition of 1 μL 0.5 M EDTA and 1 μL 0.1 mg mL^−1^ Proteinase K. The samples were incubated for 30 min at room temperature and the reaction products were run on 1% agarose gels and stained with ethidium bromide for product detection. All experiments were carried out at least in triplicate, with representative replicates shown in the figure panels.

### Purification of AcrIIC2_*Nme*_

The wild type and mutant AcrIIC2_*Nme*_ proteins were expressed in *E. coli* BL21 and purified as described previously^9^. Selenomethionine (Se-Met) labeled AcrIIC2_*Nme*_ was expressed using the methionine auxotrophic *E. coli* BL21(DE3) B834 strain cultured in M9 minimal media containing trace metals and supplemented with selenomethionine.

### Far-UV circular dichroism scans and thermal denaturation

Purified wild type and mutant AcrIIC2_*Nme*_ proteins were dialyzed into 10 mM Tris pH 7.5, 0.2 mM EDTA, 250 mM KCl. The proteins were scanned on a Jasco J-810 CD Spectropolarimeter from 200 to 260 nm. Each scan was an average of five accumulations performed at 20 nm min^−1^. For the thermal denaturation experiment, the proteins were heated at a rate of 1 °C min^−1^ from 20 to 90 °C, and the state of protein folding was assessed by absorbance at 218 nm. The assay was carried out with three biological replicates, and the standard deviation presented as the margin of error.

### Protein purification for Nme1Cas9-AcrIIC2_*Nme*_ co-crystals

Full-length genes of AcrIIC2_*Nme*_ and Nme1Cas9 were purchased from Sangon Biotech, and cloned into an expression vector pET28a-Sumo with His6-Sumo tag at the N-terminus. Mutants were constructed using a site-directed mutagenesis kit. All proteins were overexpressed in *E. coli* Rosetta (DE3) (Novagen) cells and were induced with 0.1 mM isopropyl-1-thio-β-D-galactopyranoside (IPTG) at OD_600_ = 0.6 for 12 h at 18 °C. Cells containing Nme1Cas9 were lysed by sonication in buffer containing in 20 mM Tris–HCl and 0.5 M NaCl, pH 7.5, at 4 °C. After centrifugation, the supernatant was purified by Ni Sepharose resin (GE Healthcare). Eluted Nme1Cas9 protein with His6-sumo-tag was digested with ubiquitin-like protein 1 (Ulp1) protease and dialyzed against 20 mM Tris–HCl, 0.3 M NaCl for 2 h at 4 °C to remove the His_6_-Sumo tag. Nme1Cas9 protein was further purified by Ni Sepharose column. Fractions were collected and purified on an SP column (GE Healthcare), eluting with buffer containing 20 mM Tris–HCl, pH 7.5, 1 M NaCl.

### Crystallization and structure determination of AcrIIC2_*Nme*_

Native and (Se-Met) Ni-NTA affinity-purified AcrIIC2_*Nme*_ proteins were further purified by size exclusion chromatography using a Superdex 75 column in buffer containing 20 mM Tris–HCl pH 7.5, 100 mM NaCl, 5 mM β-mercaptoethanol. Purified AcrIIC2_*Nme*_ was initially screened with 1:1 (protein:precipitant) ratio against the MCSG commercial suite and JCSG+ commercial screen using sitting drop vapor diffusion at 10 mg mL^−1^. AcrIIC2_*Nme*_ crystals were observed in 0.1 M sodium citrate, 5% propanol, and 20% PEG 4000. The crystals were further optimized with a 1:1 ratio sitting drop at 20 °C in a precipitant condition composed of 0.1 M sodium citrate, 5% propanol and 18% PEG 4000 and 15% glycerol, yielding single crystals in space group P4_1_2_1_2. Crystallographic data was collected on crystals frozen at 105 K on the 08B1-1 beam line at Canadian Light Source (CLS). Diffraction data from a total of 360 images were collected at wavelengths of 0.9795 using 1° oscillations. Data were processed with XDS package to a resolution of 2.5 Å. A complete model for AcrIIC2_*Nme*_ was solved by Se-SAD with anomalous signal from Se atoms using Phenix AutoSol. The final model was generated after several rounds of model building and refinement using Coot and PHENIX refine programs using TLS, yielding a final *R*_work_/*R*_free_ of 0.19/0.24.

### Structure determination of Nme1Cas9-AcrIIC2_*Nme*_ complex

The Nme1Cas9-AcrIIC2_*Nme*_ complex was reconstituted on ice by incubating purified Nme1Cas9 and AcrIIC2_*Nme*_ at a molar ratio of 1:10. The resulting complex was purified by gel filtration chromatography, concentrated before crystallization to an absorbance of 280 nm to ~15, as measured by Nanodrop 2000, and then set for crystal screen. The Nme1Cas9-AcrIIC2-proteolysis complex was prepared by mixing Nme1Cas9 with AcrIIC2_*Nme*_ protein at a molar ratio of 1:10 on ice for 30 min. Subsequently, the sample was purified by gel filtration chromatography. Next, α-chymotrypsin was incubated with the complex at a mass ratio of 1:500 or 1:1000 before the sample was used for crystallization.

The Nme1Cas9-AcrIIC2 complex was crystalized at 16 °C by hanging-drop vapor diffusion method. The Nme1Cas9-AcrIIC2 complex crystals were obtained by mixing 1 μL of complex solution and 1 μL of reservoir solution (0.1 M HEPES pH 7.5, 20% PEG 20,000, 0.01 M Phenol). Diffraction datasets were collected at beamline BL19U1 at Shanghai Synchrotron Radiation Facility and processed with XDS or HKL2000. The structure of the AcrIIC2-Nme1Cas9 complex was solved by molecular replacement with the AcrIIC2 dimer as the model. One Nme1Cas9-AcrIIC2 complex was identified in the asymmetric unit. The atomic model was built and refined using the programs Refmac and Phenix.

### Limited proteolysis of Nme1Cas9-AcrIIC2_*Nme*_ complex

Limited α-chymotrypsin proteolysis assays were performed at 25 °C for different times (0, 10, 30, 60 min) using proteolysis buffer 20 mM Tris pH 7.5, 300 mM NaCl. The same amount (80 μg) of purified Nme1Cas9 was used to construct complex Nme1Cas9-sgRNA (1:1.1), Nme1Cas9-sgRNA-DNA (1:1.1:1.3), Nme1Cas9-AcrIIC2 (1:4), Nme1Cas9-AcrIIC2-sgRNA (1:4:1.1), Nme1Cas9-AcrIIC2-sgRNA-DNA (1:4:1.1:1.3). The reactions were stopped by adding 2× SDS loading buffer and quenched for 10 min at 70 °C. Samples were analyzed on a 15% SDS polyacrylamide gel with Tris–Glycine buffer.

### Reporting summary

Further information on research design is available in the Nature Research Reporting Summary linked to this article.

## Supplementary Information

Reporting Summary

Supplementary Information

Source Data

## Data Availability

Structures have been deposited as PDB ID 6N05, 6JD7, 6JDJ, and 6JDX. All other datasets generated during and/or analyzed during the current study are available from the corresponding authors on reasonable request.
