# Supplementary material for: Inhibition of CRISPR-Cas9 ribonucleoprotein complex assembly by anti-CRISPR AcrIIC2
Source: Nat Commun. 2019 Jun 26;10:2806. doi: 10.1038/s41467-019-10577-3 (PMC6594998; doi:10.1038/s41467-019-10577-3)
Supplement: Supplementary file 2 — Supplementary Information [file 41467_2019_10577_MOESM2_ESM.pdf]

Supplementary Information

Inactivation of the CRISPR-Cas9 ribonucleoprotein complex by AcrIIC2

Thavalingam et al.

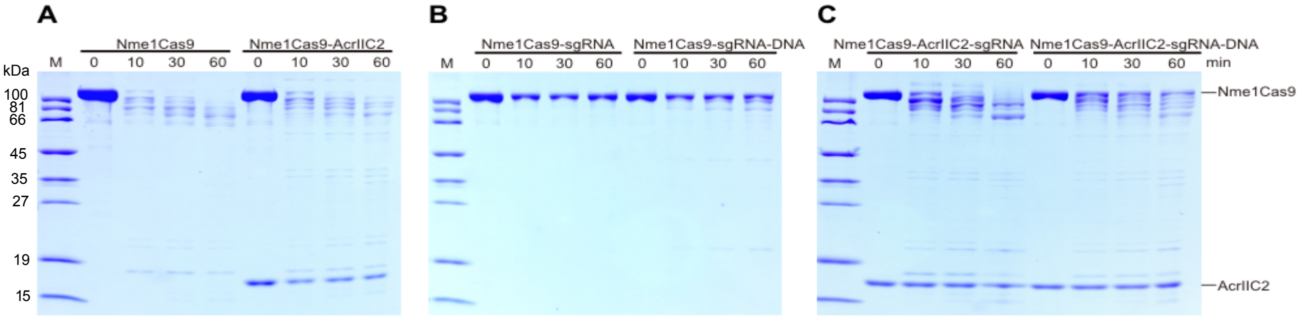

Supplementary Figure 1: Limited α-chymotrypsin proteolysis.
(A) Limited proteolysis of Nme1Cas9 with and without AcrIIC2$_{Nme}$. (B) Nme1Cas9-sgRNA with and without DNA. (C) Nme1Cas9-AcrIIC2$_{Nme}$-sgRNA with and without DNA.

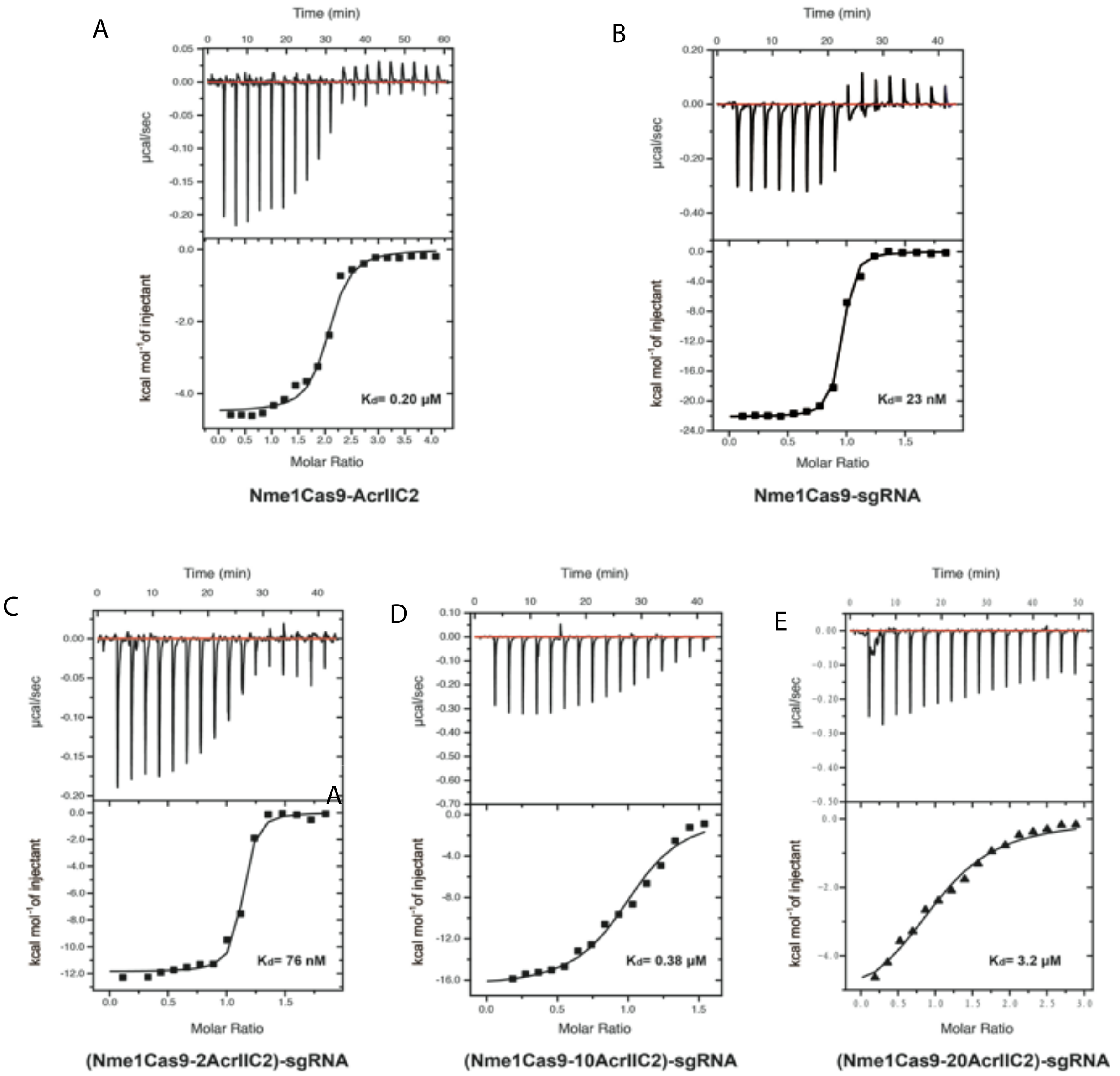

Supplementary Figure 2: Characterization of the competition of AcrIIC2*Nme* over sgRNA binding to Nme1Cas9 by isothermal titration calorimetry (ITC).
ITC measurement for the binding affinities of Nme1Cas9 toward AcrIIC2$_{Nme}$ (A) and sgRNA (B). ITC measurements for the binding affinities of (Nme1Cas9-AcrIIC2*Nme*) for sgRNA. The molar ratios of Nme1Cas9 over AcrIIC2$_{Nme}$ were set to 1:2 (C), 1:10 (D) and 1:20 (E). All ITC measurements were repeated three times with one representative experiment shown.

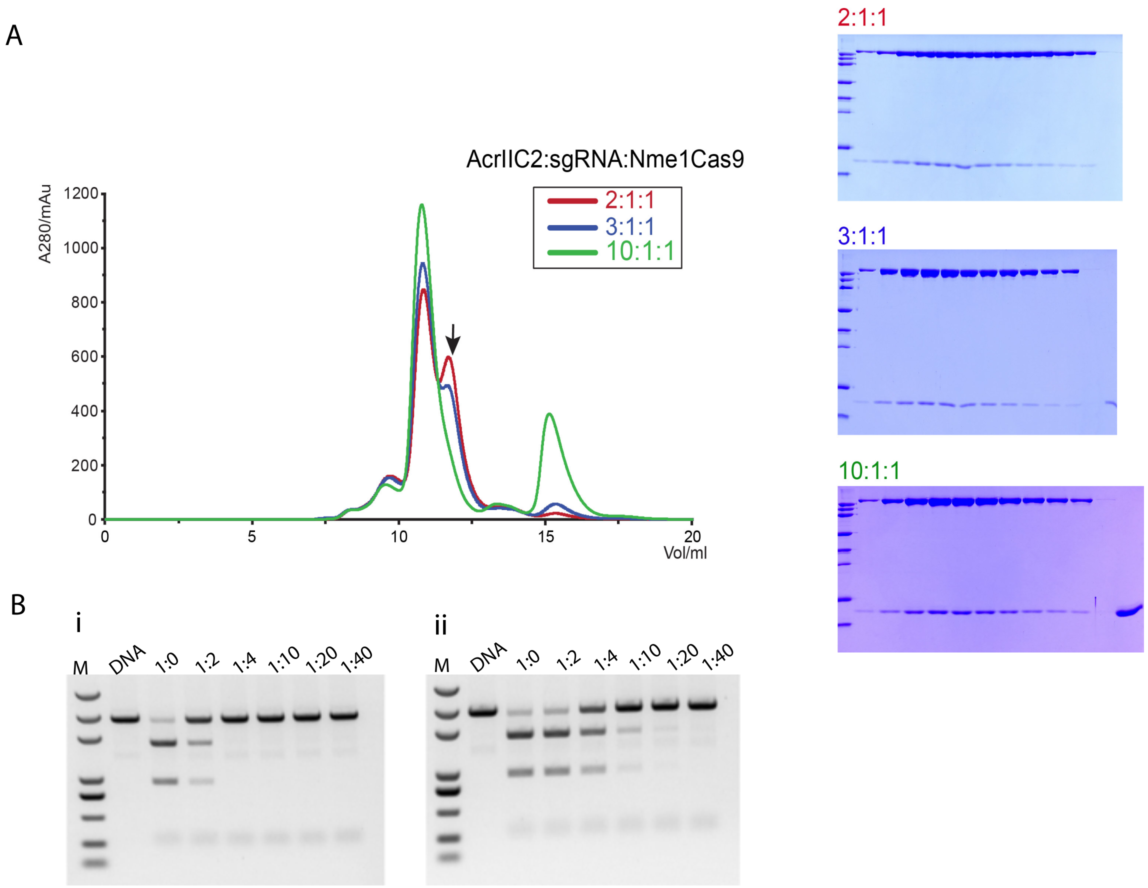

Supplementary Figure 3: Nme1Cas9-sgRNA complex formation is blocked by higher AcrIIC2$_{Nme}$ concentrations.

(A) AcrIIC2$_{Nme}$, sgRNA and Nme1Cas9 were mixed together with the molar ratio of 2:1:1, 3:1:1 or 10:1:1. The small peak indicated by the black arrow is the Nme1Cas9-sgRNA complex. No Nme1Cas9-sgRNA complex is observed when a 10-fold excess of AcrIIC2$_{Nme}$ is present. (B) Competitive cleavage assays with various molar ratios of anti-CRISPR reveals that AcrIIC2$_{Nme}$ and sgRNA compete for the binding site of Nme1Cas9. Linear plasmid DNA was used as the substrate. The molar ratio of the Nme1Cas9 and AcrIIC2$_{Nme}$ are shown above the gel. The molar ratio of Nme1Cas9 and sgRNA is 1:1.1. (i) AcrIIC2$_{Nme}$ was mixed with Nme1Cas9 and incubated for 15 min to form the AcrIIC2$_{Nme}$-NmeCas9 complex before addition of sgRNA. (ii) The AcrIIC2$_{Nme}$, sgRNA and Nme1Cas9 were added simultaneously. Nme1Cas9 and sgRNA were added at a ratio of 1:1.1 with a molar ratio of 2 to 40-fold excess of AcrIIC2$_{Nme}$, as noted above the gel.

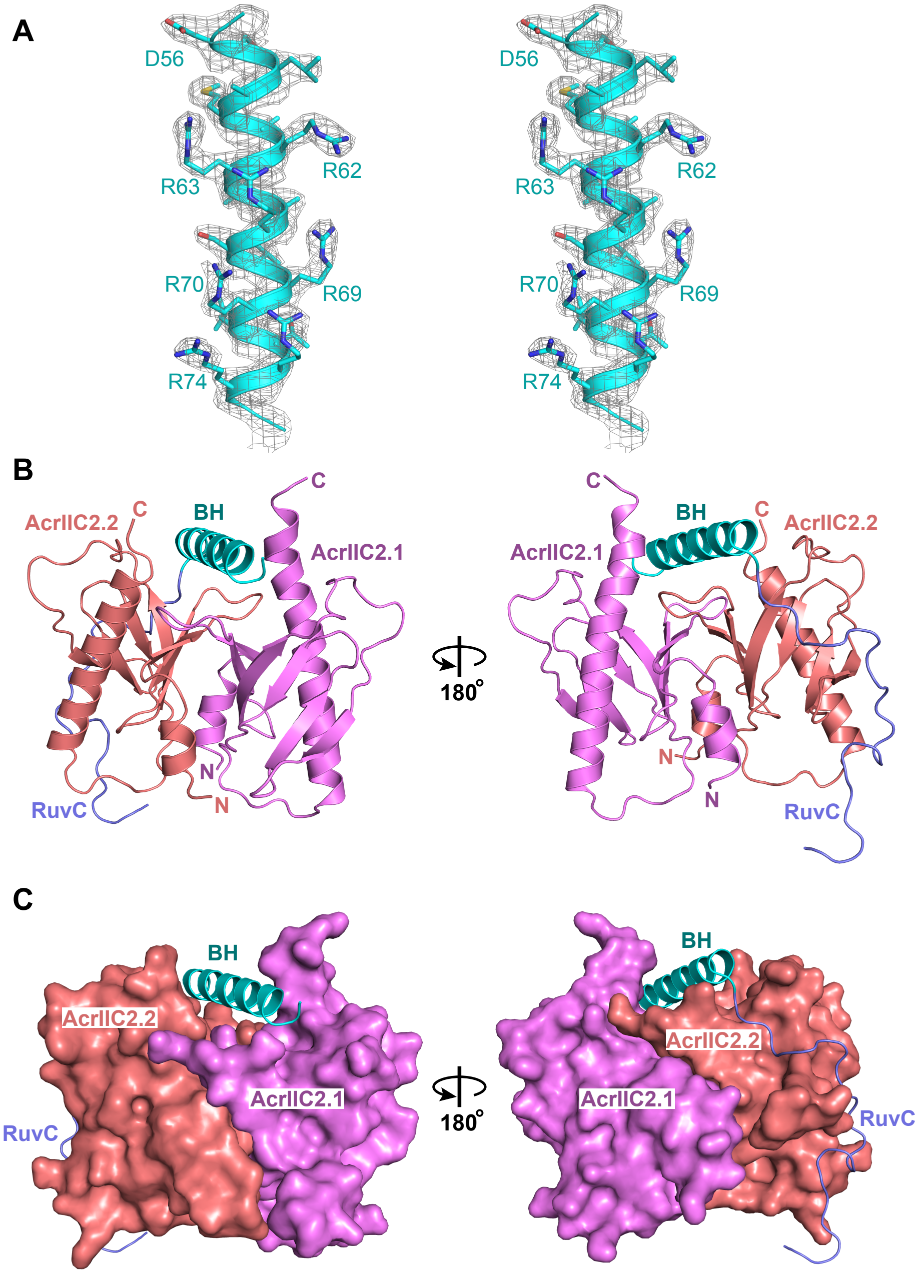

Supplementary Figure 4:. Structure of AcrIIC2*Nme*-Nme1Cas9 complex treated by α-chymotrypsin before crystallization.
(A) Stereo image of the electron density of PBD 6JDJ. Cartoon representation (B) and surface representation (C) of AcrIIC2*Nme*-Nme1Cas9 complex.

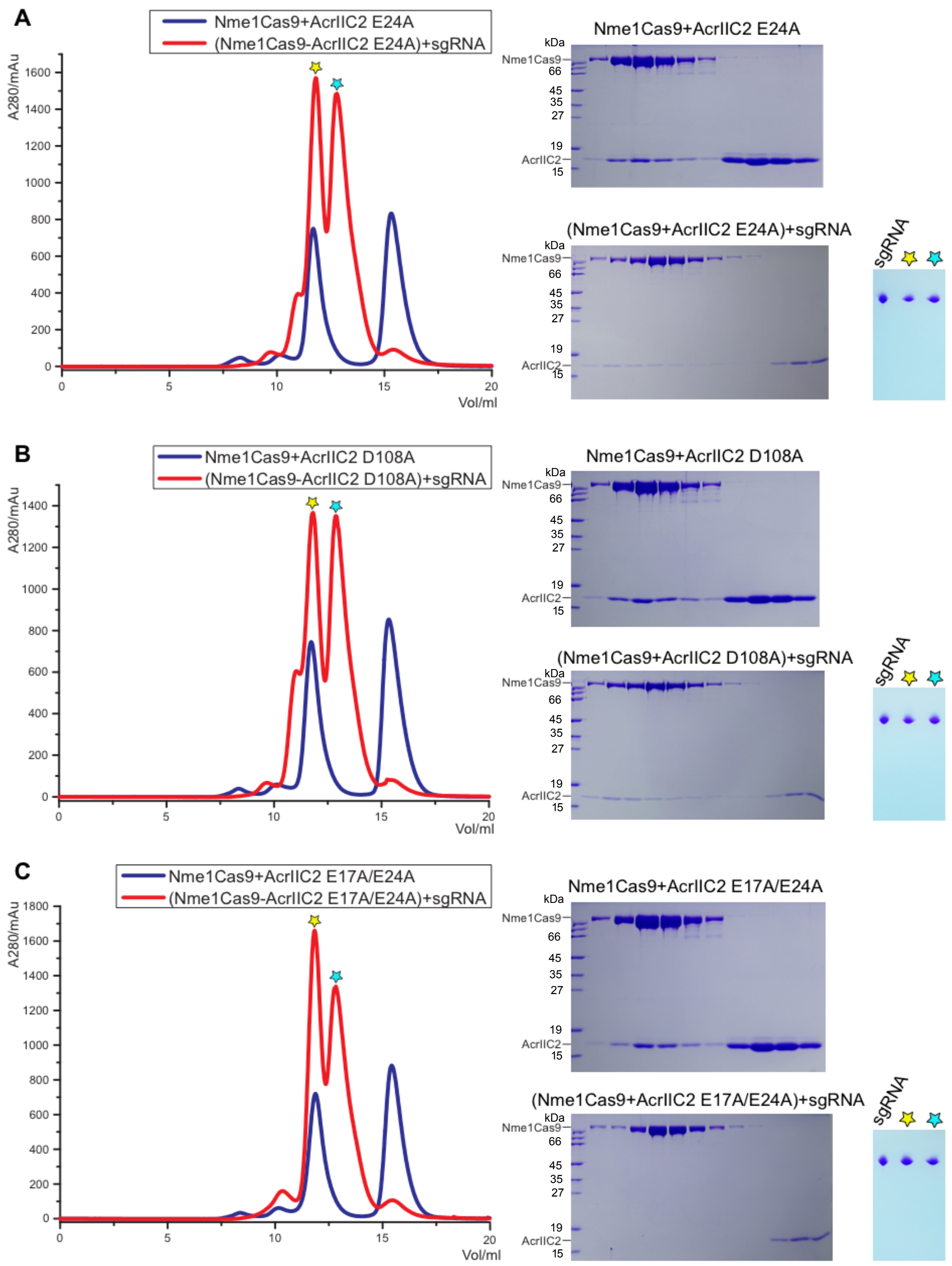

Supplementary Figure 5: Gel filtration experiments to assess the ability of AcrIIC2*Nme* mutants to interact with Nme1Cas9.

First, Nme1Cas9 incubated with $AcrIIC2_{Nme}$ mutant protein was purified by gel filtration (blue line) to get the preformed Nme1Cas9-$AcrIIC2_{Nme}$ complex. Fractions of the left peak (blue) contain the Nme1Cas9-$AcrIIC2_{Nme}$ complex. To test whether the sgRNA replaces the $AcrIIC2_{Nme}$ mutant bound to Nme1Cas9, sgRNA was added to the preformed Nme1Cas9-$AcrIIC2_{Nme}$ the complex followed by gel filtration (red line). Fractions indicated with yellow or cyan stars were analyzed on SDS-PAGE gel (left panel) and Urea-PAGE gel (right panel). The component of the peak indicated with the yellow star is Nme1Cas9-sgRNA complex, and that with the cyan star is the free sgRNA. (A) $AcrIIC2_{Nme}$ E24A mutant, (B) $AcrIIC2_{Nme}$ D108A mutant and (C) $AcrIIC2_{Nme}$ E17A/E24A mutant.

Supplementary Table 1. Primers used for construct generation

Primer	Sequence (5’-3’)
NmeCas9_Sumo_F	CGCGGATCCATGGCTGCGTTTAAACCGAAC
NmeCas9_Sumo_R	CCGCTCGAGTTAACGAACCGGCGGGCGTT
AcrIIC2_Sumo_F	CGCGGATCCATGTCTAAAAACAACATCTTC
AcrIIC2_Sumo_R	CCGCTCGAGCTAATCATCCCAACCTTCCAG
sgRNA_F	TCACTCTGCTATTTAACTTTAC
sgRNA_R	CCCAAGCTTCGCAAATCGTTT
Cje_F1	TTAAAAAATGACTCGAGTAAGGATCTCCAG
Cje_R1	GGATAAAAATGCTATGGCATAGCAAAGTG
Cje_F2	ATGCCATAGCATTTTTATCCATAAGATTAGCGG
Cje_R2	CTCTGAAAACGAAAAAACCGCCTTGCAG
Cje_F3	CGGTTTTTTCGTTTTCAGAGCAAGAGATTACG
Cje_R3	TTCTTGCCATAGATCCTTTCTCCTCTTTAGATC
Cje_F4	GAAAGGATCTATGGCAAGAATTTTGGCATTTG
Cje_R4	CTTACTCGAGTCATTTTTTAAAATCTTCTCTTTGTCTAAAC
Cje Mu targeting_F	TCCATGTTGCTAAAAAGCAAGGATGGG
Cje Mu targeting_R	ACAACCCATCCTTGCTTTTTAGCAACA
Nme1_F1	CTCGAGTAAGGATCTCCAGGCATC
Nme1_R1	GAGACCTGCCGTGGTCTCATG
Nme1_F2	CTGTTTCTCCATGAGACCACGGCAGGTCTCGTTGTAGCTCCCTTTCTCA TTTCG
Nme1_R2	TTTGATGCCTGGCAGTAAACGATGCCCCTTAAAGCAG
Nme1_F3	AAGGGGCATCGTTTACTGCCAGGCATCAAATAAAACG
Nme1_R3	TTTGAAGGCAGCCATAGATCCTTTCTCCTCTTTAGATCTTTTGAATTC
Nme1_F4	GAGGAGAAAGGATCTATGGCTGCCTTCAAACCTAATTC
Nme1_R4	GTAATGATCTCCGTAGATTTCGGCGCAGGCTTCATC
Nme1_F5	GCCTGCGCCGAAATCTACGGAGATCATTACGGCAAGAAGAATACGG
Nme1_R5	TGACAGTCTCCATATGCCCTTGCCCGCTCATCTTCC
Nme1_F6	TGAGCGGGCAAGGGCATATGGAGACTGTCAAATCCGCCAAAC
Nme1_R6	TTATTTGATGCCTGGAGATCCTTACTCGAGTTAACGGACAGGCGGGCG TTTTTTC
Nme Mu targeting_F	TCCACATCTGGTTCCAGCTCTTCAATCAG
Nme Mu targeting_R	ACAACTGATTGAAGAGCTGGAACCAGATG
Hpa_F1	ATTGGAAGTGGATAACTCGAGTAAGGATCTCCAG
Hpa_R1	CCAAGGTCGAGTCCAAGGATATAATGTAAGTTTTTATTTTCCATAGATC CTTTCTCCTCTTTAGATC
Hpa_F2	GATCTAAAGAGGAGAAAGGATCTATGGAAAATAAAAACTTACATTAT ATCCCTTGGACTCGACCTTGG
Hpa_R2	AGATCCTTACTCGAGTTATCCACTTCCAATGTTACG
Hpa_F3	GTTTTCAGAGCAAGAGATTACGCGCAGACCAAAACG
Hpa_R3	GCTATGGCATAGCAAAGTGTGACG
Hpa Mu targeting_F	TCCACTGGTTTGCCGAAAACATCGGCTGG
Hpa Mu targeting_R	ACAACCAGCCGATGTTTTCGGCAAACCAG
pMCSG7 Gib F	AACATTGGAAGTGGATAACGGATC
pMCSG7 Gib R	GGCATTGGATTGGAAGTACAGGTTC
HpaCas9 HNH Gib F	GAACCTGTACTTCCAATCCAATGCCCGTGAACTAGAAAAACGTC
HpaCas9 HNH Gib R	GGATCCGTTATCCACTTCCAATGTTTATAAATTACGTTCAATAAAGCC C

HpaCas9 PID Gib F	GAACCTGTACTTCCAATCCAATGCCAATCATGAATTTGTCCAACCTCTG
HpaCas9 PID Gib R	TCGGATCCGTTATCCACTTCCAATG
HpaCas9 RECΔBH Gib F	GAACCTGTACTTCCAATCCAATGCCGCTCGTCTGAAAAAAGC
HpaCas9 RECΔBH Gib R	GGATCCGTTATCCACTTCCAATGTTTAATGGTTTCTTCTGTTTTTTA C
HpaCas9 REC Gib F	GAACCTGTACTTCCAATCCAATGCCGCGCTTTCTCGCC
pMCSG7 mid Gib1 F	CTGATTCTGTGGATAACCGTATTACCGCC
pMCSG7 mid Gib2 R	CGGTAATACGGTTATCCACAGAATCAGG
HpaCas9 ΔHNH Gib1 R	GCGTATCATTACGATCTTTGTAAGATTTGCCAAC
HpaCas9 ΔHNH Gib2 F	ATCTTACAAAGATCGTAATGATACGCGCTACGTTGCTC
HpaCas9 ΔPID Gib F	GAACCTGTACTTCCAATCCAATGCCATGGAAAATAAAAAC
HpaCas9 ΔPID Gib R	GTTATCCACTTCCAATGTTTTAGGCTTGTGGACGATCCG
HpaCas9 ΔREC Gib1 R	TGTGGTAAGAAGTGAAACGCGACGTTGAGTTAATCGACG
HpaCas9 ΔREC Gib2 F	ACTCAACGTCGCGTTCACTTCTTACCACAGATTCCAGCTG
AcrIIC2_E17A_F	ACCCGACCATCATCCACGGTGCGGCGCGTGGTGAAAACGAT
AcrIIC2_E17A_R	ATCGTTTTCACCACGCGCCGCACCGTGGATGATGGTCGGGT
AcrIIC2_E17D_F	ACCCGACCATCATCCACGGTGACGCGCGTGGTGAAAACGATGA
AcrIIC2_E17D_R	TCATCGTTTTCACCACGCGCGTCACCGTGGATGATGGTCGGGT
AcrIIC2_E24A_F	GCGCGTGGTGAAAACGATGCGTTCGTTGTTCACACCCGTTA
AcrIIC2_E24A_R	TAACGGGTGTGAACAACGAACGCATCGTTTTCACCACGCGC
AcrIIC2_E24D_F	AAGCGCGTGGTGAAAACGATGACTTCGTTGTTCACACCCGTTA
AcrIIC2_E24D_R	TAACGGGTGTGAACAACGAAGTCATCGTTTTCACCACGCGCTT
AcrIIC2_D108A_F	GTGATCGTATCGCGGCGGATGCGCTGATGCTGAACGAAGATGC
AcrIIC2_D108A_R	GCATCTTCGTTCAGCATCAGCGCATCCGCCGCGATACGATCAC
AcrIIC2_D108E_F	GTGATCGTATCGCGGCGGATGAACTGATGCTGAACGAAGATGC
AcrIIC2_D108E_R	GCATCTTCGTTCAGCATCAGTTCATCCGCCGCGATACGATCAC
AcrIIC2_N112A_F	CGGCGGATGATCTGATGCTGGCGGAAGATGCGGCTGATCTGGA
AcrIIC2_N112A_R	TCCAGATCAGCCGCATCTTCCGCCAGCATCAGATCATCCGCCG
Nme1Cas9_R62A_F	GTGATAGCCTGGCTATGGCAGCGCGCTTAGCGCGCTCTGTA
Nme1Cas9_R62A_R	TACAGAGCGCGCTAAGCGCGCTGCCATAGCCAGGCTATCAC
Nme1Cas9_R62K_F	GTGATAGCCTGGCTATGGCAAAACGCTTAGCGCGCTCTGTACG
Nme1Cas9_R62K_R	CGTACAGAGCGCGCTAAGCGTTTTGCCATAGCCAGGCTATCAC
Nme1Cas9_R66A_F	CTATGGCACGTCGCTTAGCGGCGTCTGTACGTCGTCTGACTCG
Nme1Cas9_R66A_R	CGAGTCAGACGACGTACAGACGCCGCTAAGCGACGTGCCATAG
Nme1Cas9-R69A-F	CGCTTAGCGCGCTCTGTAGCGCGTCTGACTCGTCGTCGCGC
Nme1Cas9-R69A-R	GCGCGACGACGAGTCAGACGCGCTACAGAGCGCGCTAAGCG
Nme1Cas9_R70A_F	GCTTAGCGCGCTCTGTACGTGCGCTGACTCGTCGTCGCGCGCA
Nme1Cas9_R70A_R	TGCGCGCGACGACGAGTCAGCGCACGTACAGAGCGCGCTAAGC
Nme1Cas9_R69A_R70A_F	GTCGCTTAGCGCGCTCTGTAGCGGCGCTGACTCGTCGTCGCGCGCA
Nme1Cas9_R69A_R70A_R	TGCGCGCGACGACGAGTCAGCGCCGCTACAGAGCGCGCTAAGCGAC
Nme1Cas9_R73A_F	GCTCTGTACGTCGTCTGACTGCGCGTCGCGCGCACCGTCTGCT
Nme1Cas9_R73A_R	AGCAGACGGTGCGCGCGACGCGCAGTCAGACGACGTACAGAGC
Nme1Cas9_R74A_F	CTGTACGTCGTCTGACTCGTGCGCGCGCGCACCGTCTGCTGCG
Nme1Cas9_R74A_R	CGCAGCAGACGGTGCGCGCGCGCACGAGTCAGACGACGTACAG
Nme1Cas9_R74K_F	CTGTACGTCGTCTGACTCGTAAACGCGCGCACCGTCTGCTGCG
Nme1Cas9_R74K_R	CGCAGCAGACGGTGCGCGCGTTTACGAGTCAGACGACGTACAG
Cje_sgRNA	GUUUUAGUCCCUGAAAAGGGACUAAAAUAAAGAGUUUGCGGGACUC UGCGGGGUUACAAUCCCCUAAAACCGCUUUUUU
Nme_sgRNA	**GUUGUAGCUCCCUUUCUCAUUUCG**GAAACGAAAUGAGAACCGU UGCUACAAUAAGGCCGUCUGAAAAGAUGUGCCGCAACGCUCUGCCCC

	UUAAAGCUUCUGCUUU**AAGGGGCAUCGUUUA**
Hpa_sgRNA	GUUGUAGCUCCCUUUUUCAUUUCGCAGAAAUGCGAAAUGAAAAACG UUGUUACAAUAAGAGAAAAGAUUUCUCGCAAAGCUCUGUCCCUUGA AAUGUAAGUUUCAAGGGACAUCUUUU
